# Supplementary material for: Development of a Serum Proteomic-Based Diagnostic Model for Lung Cancer Using Machine Learning Algorithms and Unveiling the Role of SLC16A4 in Tumor Progression and Immune Response
Source: Biomolecules. 2025 Jul 26;15(8):1081. doi: 10.3390/biom15081081 (PMC12383841; doi:10.3390/biom15081081)
Supplement: Supplementary file 1 [file biomolecules-15-01081-s001.zip › biomolecules-3738110-supplementary.docx]

**Table S1.** List of differentially expressed proteins in the plasma of lung cancer patients from the GSE168198 dataset

| Protein | logFC | AveExpr | t | P.Value | adj.P.Val | B | significant |
| --- | --- | --- | --- | --- | --- | --- | --- |
| XAGE1A | −1.077 | 3.448 | −4.808 | 0.000 | 0.001 | 4.008 | Down |
| SLC16A4 | −0.589 | 2.890 | −4.603 | 0.000 | 0.001 | 3.233 | Down |
| HHLA2 | −0.667 | 3.802 | −3.996 | 0.000 | 0.005 | 1.082 | Down |
| IL2RB | −0.638 | 3.428 | −3.995 | 0.000 | 0.005 | 1.078 | Down |
| DGCR2 | −0.657 | 4.094 | −3.947 | 0.000 | 0.005 | 0.916 | Down |
| CTAG1A | −0.638 | 2.076 | −3.755 | 0.000 | 0.008 | 0.290 | Down |
| B3GALNT1 | −0.435 | 3.227 | −3.511 | 0.001 | 0.014 | −0.470 | Down |
| ASIC1 | −0.470 | 3.443 | −3.485 | 0.001 | 0.014 | −0.549 | Down |
| IRF2BP1 | −0.689 | 4.277 | −3.368 | 0.001 | 0.018 | −0.897 | Down |
| CTBS | −0.379 | 3.128 | −3.351 | 0.001 | 0.018 | −0.947 | Down |
| DKK1 | −0.409 | 3.544 | −3.294 | 0.001 | 0.018 | −1.114 | Down |
| PPM1L | −0.449 | 3.470 | −3.288 | 0.001 | 0.018 | −1.130 | Down |
| TP53 | −0.805 | 5.033 | −3.196 | 0.002 | 0.021 | −1.393 | Down |
| FYTTD1 | −0.464 | 4.134 | −3.115 | 0.002 | 0.024 | −1.618 | Down |
| XAGE3 | −0.887 | 3.475 | −3.085 | 0.003 | 0.025 | −1.700 | Down |
| PHEX | −0.449 | 3.581 | −3.054 | 0.003 | 0.026 | −1.783 | Down |
| CYP4F11 | −0.381 | 3.656 | −3.005 | 0.003 | 0.028 | −1.916 | Down |
| ANXA1 | −0.327 | 2.657 | −3.003 | 0.003 | 0.028 | −1.920 | Down |
| PPP4R1 | −0.595 | 4.354 | −2.953 | 0.004 | 0.030 | −2.052 | Down |
| CFHR5 | −0.384 | 3.534 | −2.919 | 0.004 | 0.032 | −2.142 | Down |
| CAGE | −0.519 | 2.294 | −2.784 | 0.006 | 0.045 | −2.486 | Down |
| EIF6 | −0.325 | 3.033 | −2.725 | 0.007 | 0.048 | −2.632 | Down |
| P2RX5 | −0.459 | 4.223 | −2.688 | 0.008 | 0.050 | −2.722 | Down |

**Table S2.** Evaluation of the four lung cancer diagnostic models in the training and testing datasets

| Protein | AUC | Accuracy | Sensitivity | Specificity |
| --- | --- | --- | --- | --- |
| DKK1 | 0.685 | 0.700 | 0.750 | 0.650 |
| MYC | 0.640 | 0.633 | 0.583 | 0.683 |
| TP53 | 0.636 | 0.633 | 0.517 | 0.750 |
| MUC1 | 0.634 | 0.650 | 0.633 | 0.667 |
| SOX2 | 0.619 | 0.633 | 0.583 | 0.683 |
| EZR | 0.613 | 0.583 | 0.733 | 0.433 |
| HDAC1 | 0.609 | 0.625 | 0.817 | 0.433 |
| YAP1 | 0.592 | 0.642 | 0.850 | 0.433 |
| HER2 | 0.565 | 0.617 | 0.700 | 0.533 |


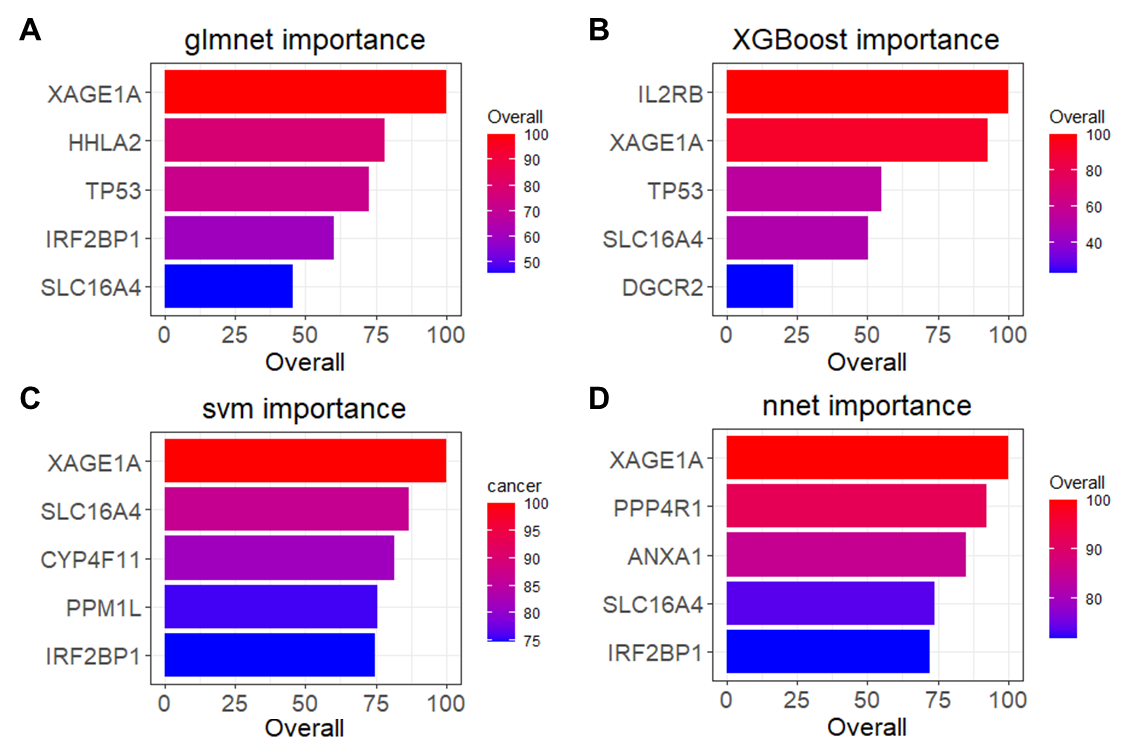


**Figure S1.** Bar plots showing the top 5 most important proteins in each of the four models. (A) glmnet, (B) XGBoost, (C) svm, (D) nnet.


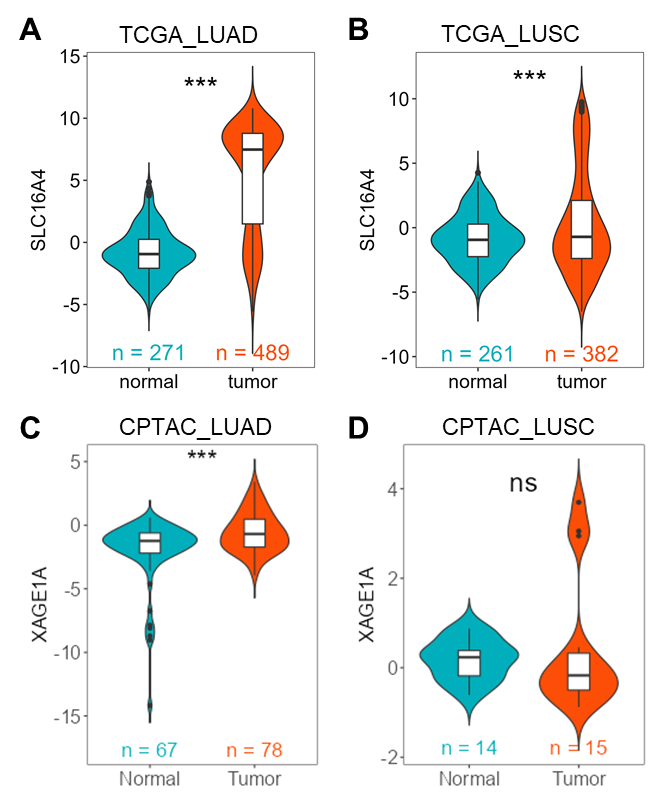


**Figure S2**. XAGE1A is significantly upregulated in lung cancer. The mRNA expression differences of XAGE1A between lung cancer and normal tissues in the TCGA LUAD (A) and LUSC (B) datasets and in protein expression in the CPTAC LUAD (C) and LUSC (D) datasets. Compared with normal group, * *p* < 0.05, ** *p* < 0.01, *** *p* < 0.001. TCGA, The Cancer Genome Atlas. CPTAC, Clinical Proteomic Tumor Analysis Consortium. LUAD, Lung Adenocarcinoma. LUSC, Lung Squamous Cell Carcinoma.
